# Effect of the enhancing nutrition and antenatal infection treatment (ENAT) intervention on birth weight in Ethiopia: a cluster randomized controlled trial

**DOI:** 10.1186/s12884-023-05912-y

**Published:** 2023-08-29

**Authors:** Y Mekonnen, E Wolde, A Bekele, Z Mehari, S Abebe, T Hagos, Y Tadesse, T Taye, G Asire, T Nigatu, S Kumar, S Girma, M Salasibew

**Affiliations:** 1 Mela Research, P.O. Box 34422, Addis Ababa, Ethiopia; 2 Children’s Investment Fund Foundation (CIFF), Addis Ababa, UK; 3 Jhpiego Ethiopia, Addis Ababa, Ethiopia; 4 John Snow Inc., Addis Ababa, Ethiopia

**Keywords:** Ethiopia, Birth weight, Antenatal care, Point-of-care testing, ENAT, CRCT

## Abstract

**Background:**

The Enhancing Nutrition and Antenatal Infection Treatment (ENAT) intervention was implemented in Ethiopia to improve newborn birth weight (BW) by strengthening the contents and quality of antenatal care (ANC), especially point-of-care testing for maternal infections. This study examined the effect of the ENAT intervention on birth weight.

**Methods:**

We conducted a cluster randomized controlled trial of 22 clusters (health centers), randomized equally between 11 intervention and 11 control clusters. This study enrolled and followed pregnant women from ANC booking to the end of pregnancy or loss to follow-up. The primary outcome was mean BW, and the incidence of low birth weight (LBW) was the secondary outcome. We presented univariate comparisons of outcomes between the intervention and control arms for mean BW and LBW. Multilevel analyses using random effects models were performed to adjust for clustering and individual-level covariates.

**Results:**

We enrolled and followed up 4,868 and 4,821 pregnant women in the intervention and control arms, respectively, from March 2021-July 2022. During follow-up, 3445 pregnant women in the intervention and 3192 in the control delivered in the health centers, and BW measurements of their babies were recorded within 48 h. The mean BW was 3,152 g (standard deviation (SD) = 339.8 g) in the intervention and 3,044 g (SD = 353.8 g) in the control arms (mean difference, 108 g; 95% confidence interval (CI): 91.3-124.6; P = 0.000). Adjusting for clustering and several covariates, the mean BW remained significantly higher in the intervention arm than in the control arm (adjusted ß coef., 114.3; p = 0.011). The incidence of LBW was 4.7% and 7.3% in the intervention and control arms, respectively. The adjusted risk of LBW was significantly lower by 36% in the intervention arm than in the control arm (adjusted relative risk, 0.645; p = 0.027).

**Conclusion:**

This study provided sufficient evidence of the effectiveness of the ENAT intervention in improving birth weight in the study population. The intervention demonstrated that an increase in birth weight can be attained by availing point-of-care testing, strengthening infection prevention, and maternal nutrition within the ANC platform of public health facilities in a low-income setting.

**Trial Registration:**

Registered at Pan African Clinical Trial Registry (PACTR) database dated 09/05/2023, https://pactr.samrc.ac.za/TrialDisplay.aspx?TrialID=25493. The unique identification number for the registry is PACTR202305694761480.

## Background

Globally, approximately one in seven newborns is born with LBW [1]. These babies are more likely to die during their first month of life or face lifelong consequences [2–5]. In Ethiopia, LBW continues to pose a significant public health challenge, although there is no reliable prevalence estimate to assess its current extent. The data available from small studies and national surveys indicate that LBW rates in the country fall within the range of 6–29%, with average BW ranging from 2800 to 3200 g [6–12].

The World Health Organization (WHO) acknowledges the LBW rate as one of the key newborn health indicators prioritized by the global community. It serves to provide concise information on health situations, trends, and responses at both national and global levels [13]. There is also a global nutrition target to achieve a 30% reduction in the number of infants born with a weight lower than 2500 g by the year 2025, which would translate into a 3% relative reduction per year between 2012 and 2025 and a reduction from approximately 20 million to approximately 14 million infants with low weight at birth [14]. Recent global estimates of LBW have shown some progress in reducing the risk of LBW at the global level, although there is still a long way to go to meet the global nutrition target [15].

There are evidence-based interventions available to reduce LBW. Recommended actions include promoting access to nutritious, safe, affordable, and sustainable diets and delivering micronutrient supplementation for women [3]. Early and continued access to high-quality antenatal care (ANC) is a gateway to preventing infectious diseases and managing pregnancy-associated health conditions and birth outcomes [16]. ANC provides a platform for pregnant women to receive services that are critical for their health and that of their fetuses, including early detection of danger signs, essential preventive intervention, counseling and treatment services. The comprehensive WHO guidelines on focused ANC packages identified the services to be given to pregnant women during their ANC visits, including tracing maternal disease history; examinations of blood pressure, anemia, and fetal movement; screening and tests; treatment of syphilis and bacteriuria if indicated; preventive measures such as tetanus toxoid immunization and iron and folic acid supplementation; and overall health education [17].

The Ethiopian Ministry of Health has identified ANC among the priority interventions in its Health Sector Transformation Plan to prevent poor maternal and newborn outcomes, including LBW [18]. Ethiopia has also adopted the WHO recommendation of focused ANC that encompasses interventions to improve pregnancy outcomes, such as maternal assessment, preventive measures, maternal nutrition-related assessment, and intervention, among others [19]. Despite this, however, most public health facilities in the country reported suboptimal ANC services, and pregnant women attending ANC do not always receive the essential ANC services as stipulated in the guidelines [20–23].

The Enhancing Nutrition and Antenatal Infection Treatment (ENAT) intervention project was designed to improve newborn birth weight by strengthening the availability of point-of-care tests in public health centers for the prevention and treatment of infections during pregnancy [24]. The intervention was implemented in 65 rural health centers and their catchment communities in selected Woredas (districts) of the Amhara and Oromia regions of Ethiopia. Using a two-arm parallel cluster randomized controlled trial (CRCT), we evaluated the impact of the ENAT intervention on birth weight, and this paper presents the findings of the study.

## Methods

### Trial design

We implemented a two-arm parallel cluster randomized controlled trial (CRCT), in which a cluster was defined as a health center (HC). According to the Ethiopian Ministry of Health classification of health facilities, a primary health care unit (PHU) is composed of a health center and five satellite health posts (HPs). A health center provides services to approximately 25,000 people altogether and provides both preventive and curative services. This study had two arms: intervention and control, and the clusters were randomized and assigned in a 1:1 ratio to the two arms. In the control health centers, the pregnant women received the routine Ethiopian government (EG) ANC, while in the intervention health centers, they received the ENAT intervention on top of the usual EG routine services. This study enrolled and followed up pregnant women from ANC booking to the end of pregnancy or loss to follow-up. As part of enrollment, this study collected baseline enrollment information via individual interviews at ANC booking, and after enrollment, data were extracted regularly from the routine ANC and delivery registration books.

### ENAT intervention

The ENAT intervention was implemented in 65 health centers and their catchment communities in selected Woredas (districts) of the Amhara and Oromia regions of Ethiopia. ENAT was a bundle of interventions that included strengthening/introducing point-of-care diagnostics technology for screening of maternal infections and treatment, training of health care providers on basic antenatal care service provision including comprehensive counseling, screening for infection and treatment, and provision of preventive care, and strengthening/introducing a system for tracking pregnant women to verify and support continuity of care, i.e., adherence to ANC services. The intervention also strengthened existing community activities to identify pregnant women and improve the early initiation of pregnant women to ANC services. The main intervention activities can be summarized as follows:

- *Clinical skills strengthening training*: The training focused on ensuring the improvement of the quality of ANC services that included ANC skills-building trainings for midwives, nurses, and laboratory technicians.
- *Point-of-care (POC) testing training*: ENAT strengthened the availability and capacity of PoC tests, including asymptomatic bacteriuria (ASB), syphilis, hemoglobin and others. Laboratory technicians and technologists from selected universities and regional laboratories conducted periodic supportive supervision and quality assurance in the project health centers.
- *Availing essential equipment and supplies*: The project health centers were supplied with necessary urine dipsticks, syphilis tests, hemoglobin tests, and essential ANC equipment, including a BP apparatus and weighing scales. As part of the regular monitoring of health facilities, the ENAT field team conducted a periodic assessment of essential commodities and supplies to inform project-supported procurement.
- *Improving nutrition intervention*: ENAT introduced different strategies to prevent nutritional disorders during pregnancy. Some of the interventions were providing screening pregnant mothers for anemia and malnutrition using mid-upper arm circumference (MUAC) measurement. A tracking mechanism was used to monitor the provision of deworming and Iron and Folic Acid (IFA) tablets. Additionally, essential job aids for nutritional counseling and gestational weight monitoring were developed and distributed.
- *Mentoring and supportive supervision* were critical components of the ENAT to ensure the quality and completeness of the project implementation. A comprehensive checklist was developed and used to guide mentoring and supervision activities. During mentoring and supervision, detailed assessments were made on providers’ competencies and availability of supplies and commodities, in addition to comprehensive technical support to the health centers based on the identified gaps. The project tasked full-time ENAT field workers to provide regular mentorship and supportive supervision, occurring at least twice per quarter.

### The standard ANC package in the health centers

The standard ANC package adopted by the Ethiopian government is based on the 2002 focused antenatal care guidelines and approach of the World Health Organization. The routine ANC guideline includes various components such as identifying and treating diseases, early detection of potential complications that could impact pregnancy outcomes, and providing prophylaxis and treatment for conditions like anemia, malaria, sexually transmitted infections (STIs) including HIV, urinary tract infections, and tetanus. Moreover, the routine ANC entails offering timely guidance and advice to pregnant women on topics like birth preparedness, nutrition, immunization, personal hygiene, and family planning. It also involves counseling expectant mothers on recognizing danger signs and symptoms that necessitate immediate assistance from a healthcare professional [19].

### Outcome measures

The primary and secondary outcome measures of this study were mean BW and LBW, respectively. LBW is defined as birth weight values less than 2500 g.

### Sample size

The sample size for this study was powered to detect a minimum 70-gram increase in mean birth weight in the intervention area compared to the control. The parameter inputs for the sample size included an expected baseline birth weight of 3000 g, a value taken from available published studies [12], anticipated effect size (BW = 70 g), study power (0.8), confidence level of the test (0.05), and interclass correlation (0.006). In most CRCT studies, Interclass Correlation Coefficient (ICC) are between 0.001 and 0.05 [25]; although such values may seem small, their effects on power and type I error rates can be very large [26, 27]. We used the STATA inbuilt program—*clustersampsi* [28]—to estimate the sample size requirements for this study, and the resulting sample size for the CRCT given the aforementioned parameters was 11 clusters per arm and an average cluster size of 184 live institutional births (with BW measured). Assuming substantial loss to follow-up (LTFU) occurring due to home deliveries, miscarriage, stillbirth, and change of residence (i.e., altogether estimated at 41%), we needed to enroll 311 pregnant women per cluster and 3421 per arm to achieve the desired sample size for the number of births with birth weight. This sample size was also powered to detect a 30% reduction in LBW in the ENAT intervention arm compared to the control arm. This sample size was later revised in the middle of this study to capture more pregnant women who initiated first ANC before 16 weeks of gestational age, and this sample size adjustment was made because early ANC is often regarded as a key success metric. Accordingly, we increased the trial sample size by an additional 1463 pregnant women per arm, resulting in a total trial sample size of 4884 pregnant women per arm. As a result of the increased sample size, the study participants’ enrollment and follow-up time was prolonged by approximately four months.

### Cluster randomization

The unit of randomization was a health center (cluster). The ENAT intervention was implemented in three zones of Ethiopia, namely, *Arsi (Oromia region), South Gondar (Amhara region), and West Gojam (Amhara region)*. At the start of the ENAT implementation, there were 328 health centers in these zones, and of these, 22 were randomly selected using simple random sampling and then randomized equally (n = 11) between the intervention and control arms. The ENAT intervention was implemented in 65 health centers across the three zones, with 11 of them serving as the designated study health centers. Stratified randomization was employed by zone to assign the clusters between the two arms. This was to take into account any zone-based variation between the two arms, including the sociodemographic and health contexts, ANC utilization rate, quality of ANC services, LBW rate, and other potential confounders. This helped to alleviate any possible imbalances between the intervention and control arms. In each stratum (zone), we assigned the clusters to the two arms using a simple unrestricted randomization method.

### Participant enrollment and follow-up

Enrollment in this study followed the EG routine ANC booking and registration procedure in the health centers. Health center midwives collected the required information from pregnant women at ANC booking according to the national ANC registration guidelines. After the routine ANC booking and initial assessment by the midwives, the pregnant women were invited to participate in this study. We recruited and deployed dedicated study midwives in each of the study health centers who enrolled pregnant women, interviewed them (at enrollment), and extracted study participants’ ANC service- and delivery-related data from the ANC and delivery registration books. The pregnant women were given complete information as to the objective of this study and their benefits/risks, and only after providing their consent were they enrolled in this study. The eligibility criteria for the pregnant women to be included in this study were that they must be enrolled in this study at their first ANC booking and had a confirmed positive pregnancy test. There was no limit on gestational age at enrollment to participate in this study. The enrollment characteristic information of the pregnant women collected included personal identifiers, residence, gestational age, gravida, number of children ever born, education, and household assets (proxy to economic status), among a few others. During follow-up, the ANC and delivery-related data of the study participants were extracted regularly from the routine ANC and delivery registration books. A woman-baby dyad (pair) was considered lost to follow-up if a woman did not come to the facility for a follow-up visit, changed her residence, died, miscarried, or aborted. Home deliveries without birthweight data were also considered lost to follow-up. The CRCT study, including enrollment and follow-up, was conducted during the period from March 2021 to July 2022.

Data quality assurance and supervision were an integral part of this study, and these in particular focused on the accuracy, completeness, and recording of birth weight. We provided a highly accurate, new digital newborn weight scale (known as *Dr. Care*) to the 22 study health centers of both arms. The weight scales were placed on flat and neat surfaces in each of the study health centers. We also provided practical in-service training to the facilities midwives/nurses on how to use the new scale, how and when to calibrate it, and how to properly record birth weight data and transfer the data onto the delivery registration book. They were also informed to calibrate the scales every day using some standard weights (e.g., 2000 g). During the study period, the quality of the birth weight data was monitored closely by the study midwives who were stationed in the health centers by checking the functionality of the weight scales, calibrating the scales as needed, and continuously working with the facilities midwives/nurses to maintain the accuracy and completeness of the birth weight data.

### Statistical analysis

The analysis of the outcome was primarily by intention-to-treat. An intention-to-treat analysis is a method for analyzing results in a prospective randomized study where all randomized participants are included in the statistical analysis and analyzed according to the group they were originally assigned, regardless of what treatment (if any) they received [29]. The primary aim of this analysis was to establish whether there was a statistically significant increase in mean BW in the ENAT intervention arm compared to the control. As a secondary outcome, the analysis also tested whether the prevalence of LBW, defined as BW less than 2500 g, was significantly lower in the intervention arm than in the control arm. We presented univariate comparisons of mean BW and LBW between the intervention and control arms for the overall sample and presented subgroup analyses of differences in mean BW or LBW between the two arms by newborn sex, mother’s age, and gestational age at first ANC booking. The cluster-weighted chi-square test was used to compare categorical variables between the two arms. This test was adjusted for the effect of clustering, pooled across the study arms. To compare continuous variables between the study arms, we used a cluster-adjusted t test. Multilevel analyses using random effects models were performed to adjust for clustering and selected individual-level covariates. A p value below 0.05 suggested statistical significance. Participants’ enrollment bias, which could arise from post randomization selection bias, can be one of the problems of cluster randomized trials, and this was tested by comparing baseline (enrollment) characteristics of study participants. Four groups of pregnant women were compared based on their enrollment characteristics: (a) pregnant women with outcomes (intervention arm); (b) pregnant women who were lost to follow-up (intervention arm); (c) pregnant women with outcomes (control arm); and (d) pregnant women who were lost to follow-up (control arm). We also compared key ANC services and interventions between the two arms. All data analyses were conducted in STATA 17(Stata Corporation, College Station, TX, USA).

## Results

### Participant flow diagram

**Fig. 1:**
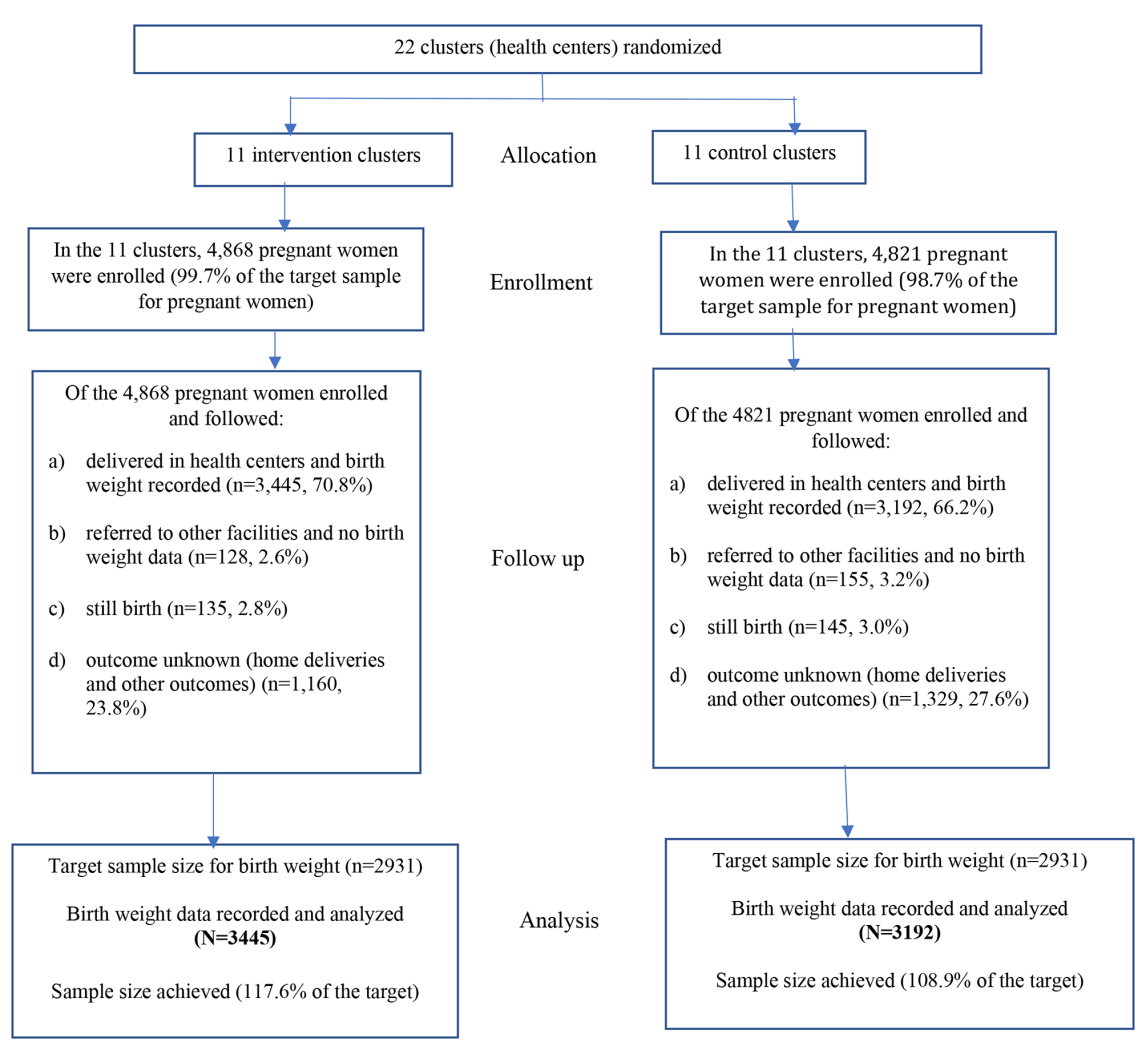
Participants’ enrollment and follow-up by study arm

The study participants’ flow diagram is shown in Fig. 1. During the period from March to November 2021, 4,868 pregnant women were enrolled in the intervention arm, and 4,821 in the control. This represented 99.7% and 98.7% of the target sample size, respectively. The pregnant women were followed until the end of pregnancy or loss to follow-up, and during follow-up that spanned through the end of July 2022, of those enrolled in the intervention arm, 70.8% (n = 3445) delivered live births in the health centers they were enrolled in and had BW records. In the control arm, the corresponding figure was 66.2% (n = 3192). There were referrals of pregnant women to other facilities that comprised 2.6% (n = 128) in the intervention and 3.2% (n = 155) in the control arms, and these pregnant women did not have BW data. Based on records of the health centers, 2.8% (n = 135) of the pregnant women enrolled in the intervention arm and 3% (n = 145) in the control arm had stillbirths. Our follow-up data also showed that home deliveries were common and that the babies were not weighed at birth. The proportions of pregnant women who delivered in their homes were 23.8% (n = 1160) and 27.6% (n = 1329) in the intervention and control arms, respectively. The final analysis this study was based on those babies with BW, comprising 70.8% (n = 3,445) births in the intervention and 66.2% (n = 3192) in the control. The sample sizes achieved for birth weights in both study arms were slightly larger than the required sample size for this study.

### Comparison of enrollment characteristics

Although this study achieved more than the anticipated sample size for BW in both study arms, 29.2% of the pregnant women in the intervention arm and 33.7% in the control arm did not have BW data and were thus considered lost to follow-up. We compared selected characteristics of the participants across the four groups: Intervention-with outcome; Intervention- LTFU; Control-with outcome; and Control-LTFU. As shown in Table 1, in both study arms, the participants with the outcome were broadly similar to those lost to follow-up in terms of several enrollment characteristics, including women’s age, education, household wealth, fertility profiles, maternal nutritional status, and gestational age at the first ANC visit. We presented cluster-adjusted p values for each of the enrollment characteristics to compare across the four groups. The mean age at enrollment compared well across the four groups, ranging from 25.7 to 26.4 years (p = 0.491). The participants in the four groups, on average, had 2.8 pregnancies, and there were no statistically significant differences in the number of pregnancies across the four groups (p = 0.576). There were no significant variations in women’s education and household wealth across the four groups. The timing of the first ANC visit was trichotomized, with the first category being having the first ANC visit in the first trimester of pregnancy (< 16 weeks). As the data show, the proportions that initiated the first ANC visit in the first trimester of pregnancy were comparable across the four groups and ranged between 33.3% and 41.5% (p = 0.067). The nutritional status of women was assessed using mid-upper arm circumference (MUAC), and a MUAC value of less than 23 cm is indicative of malnutrition. The proportion categorized as being malnourished using MUAC cutoff values was almost similar in the four groups (P = 0.156), ranging from 12.1 to 13.6% across the four groups.

**Table 1:** Enrollment characteristics of those with the outcome (birth weight recorded) vs. those lost to follow-up by study arm

	Intervention: with outcome	Intervention: LTFU	Control: with outcome	Control: LTFU	P value Ϯ
	N = 3445	N = 1423	N = 3192	N = 1229	
Mean age of women (95% CI)	25.7(25.4–25.9)	26.1(25.9–26.3)	26.4(26.1–26.6)	26.0(25.8–26.2)	0.491
Mean number of pregnancies (95% CI)	2.8(2.6–2.9)	2.8(2.7–2.9)	2.7(2.6–2.8)	2.7(2.6–2.8)	0.576
No education	47.8	45.9	43.5	41.8	0.821
Education: grades 1–6	19.4	19.0	16.9	16.6	0.996
Education: grades 7–8	14.5	13.8	13.4	14.1	0.872
Education: grades 9+	18.3	21.3	26.3	27.6	0.889
Wealth quintiles: Lowest	23.4	27.5	15.4	15.3	0.408
Wealth quintiles: Second	23.2	19.3	17.5	18.5	0.995
Wealth quintiles: Middle	17.5	16.0	22.1	24.6	0.924
Wealth quintiles: Fourth	18.5	18.9	21.7	20.9	0.793
Wealth quintiles: Highest	17.3	18.4	23.3	20.8	0.388
First ANC visit: <16 weeks	37.4	41.5	33.3	37.0	0.067
First ANC visit: 16–27 weeks	47.3	45.1	53.4	49.9	0.061
First ANC visit: >27 weeks	15.3	13.5	13.4	13.1	0.454
% Malnourished (MUAC < 23 mm)	13.6	12.8	12.1	13.4	0.156

Ϯ *P value compare the four groups; P value adjusted for clustering*

### Comparison of the coverage of ANC services

This analysis is restricted to those participants with recorded outcomes, i.e., with BW measurements. Included in the present analyses were those services that are part of the routine ANC services of the public health facilities in Ethiopia. A total of 14 ANC services that are routinely registered in the ANC book were included in this analysis and compared between the intervention and control arms (Table 2). For each ANC service, we defined service coverage if the service was provided to the pregnant women at least once during pregnancy. A summary composite score that combined the 14 services was calculated that categorizes the study participants as receiving low (< 7 services), medium (7–9 services), and high (10–14 services). The summary score revealed that pregnant women in the intervention arms were significantly more likely than those in the control to have received most of the ANC services, with over 82% of them categorized as scoring high in the composite coverage score. The corresponding proportion that scored high in the control arms was significantly lower at 54.2% (p = 0.009). Coverage for individual interventions also showed significantly higher percentages in the intervention arm than in the control arm for eight out of the 14 components. The intervention-control coverage gap was the highest for deworming at 41.5% (49.6% intervention vs. 8.1% control), followed by the hemoglobin test at 36.3% (92.9% vs. 56.6%); asymptomatic bacteriuria (ASB) at 32.3% (71.1% vs. 38.8%); urinalysis for protein at 27.6% (95.5% vs. 67.9%); and syphilis test at 22.5% (94.2% vs. 71.7%), among a few others.

**Table 2:** Coverage of selected ANC services (received/provided at least once during ANC visits) among pregnant women with the outcome (birth weight recorded) by study arm

	Intervention	Control	P value Ϯ
	N = 3445	N = 3192	
**Mean exposure time in weeks (95% CI)**	20.2 (19.8–20.5)	20.3(19.9–20.6)	0.2059
**ANC services (%)**			
Women weighed	97.7	80.6	0.044
MUAC measured	96.1	82.4	0.050
Blood pressure measured	93.6	92.6	0.838
Hemoglobin tested	92.9	56.6	0.009
Screened for asymptomatic bacteriuria (ASB)	71.1	38.8	0.027
Urinalysis for protein	95.5	67.9	0.017
Syphilis test	94.2	71.7	0.033
Urinalysis for sugary test	58.7	42.7	0.039
Blood groping RH	90.0	81.8	0.481
HIV test	95.7	94.4	0.743
Hepatitis test	81.9	83.9	0.855
Deworming tablet given	49.6	8.1	0.000
Tetanus toxoid injection (TTI) given	98.2	93.8	0.186
Iron folate given	89.1	86.4	0.757
**Summary score: (based on 14 services)**			0.000
Low: received: < 7 services	2.4	11.8	
Medium: received: 7–10 services	15.1	34.0	
High: received: 10–14 services	82.5	54.2	
Mean score (95% CI)	12.0(11.9–12.1)	9.8(9.7–9.9)	0.009

Ϯ *P value compare intervention and control; P value adjusted for clustering*

### Comparison of main outcomes

#### Mean birth weight (BW)

The primary outcome of interest of this study was the mean BW, and we present the mean BW for the overall sample and stratified by newborn sex, mother’s age, and gestational age at the first ANC visit. As shown in Table 3, the mean BW was significantly higher at 3152 g in the intervention arm compared to 3044 g in the control, and the mean difference in BW between the two arms was 108.0 g (95% CI: 91.3-124.6; P = 0.000). The subgroup analyses by newborn sex, mother’s age and gestational age at the first ANC visit found consistently significantly higher mean BW values in the intervention arm than in the control arm across the different subgroups. For the male babies, the mean BW was significantly higher by 106.0 g (95% CI: 82.7-129.3; P = 0.038) in the intervention arm than in the control arm. An almost similar increase in mean BW of 110.2 g (95% CI: 86.3–134.0; p = 0.014) can be seen for female babies in the intervention arm than in the control. Mother’s age was categorized into four groups, and we compared the mean BW between the two arms for each age group. Overall, we found that the mean BW was significantly higher in the intervention arm than in the control arm across all age groups. We dichotomized gestational age at the first ANC visit as less than 16 weeks and 16 weeks or higher. For pregnant women who initiated the first ANC visit before 16 weeks, the mean BW was significantly higher at 3136.5 g in the intervention arm than 3018.3 g in the control (p = 0.017). Similarly, for pregnant women who had their first ANC booking later than the first trimester, the mean BW was significantly higher in the intervention (3165 g) than in the control (3057.9 g) (P = 0.033).

**Table 3:** Unadjusted mean birth weight (BW) and standard deviation (SD) and the absolute differences in mean birth weight (and 95% CI) for the total sample and according to newborn sex, mother’s age and gestational age at first ANC visit, by study arm

	Intervention		Control		Absolute difference in mean birth weight (p value Ϯ)	95% Confidence Interval	
	**n**	**mean BW(SD)**	**n**	**mean** **BW(SD)**		**Lower**	**Upper**
All sample	3445	3152.1 (339.8)	3192	3044.1(353.8)	108.0 (p = 0.000)	91.3	124.6
Newborn sex							
Male	1773	3168.1(342.9)	1647	3062.1(352.2)	106.0 (p = 0.018)	82.7	129.3
Female	1672	3135.2(335.7)	1545	3025.0(354.5)	110.2 (p = 0.014)	86.3	134.0
Age of mother							
15–19	218	3116.8(320.3)	241	3014.4(368.8)	102.4(p = 0.027)	38.8	166.1
20–24	1056	3128.4(333.5)	959	3045.3(361.6)	83.1(p = 0.047)	52.6	113.4
25–34	1827	3163.1(335.9)	1709	3052.7(340.9)	110.4(p = 0.014)	88.0	132.7
35–49	344	3188.0(383.1)	282	3012.0(387.2)	176.0(p = 0.008)	116.1	237.6
Gestational age at first ANC visit							
< 16 weeks	1249	3136.5(353.4)	1054	3018.3(371.7)	118.2(p = 0.017)	88.6	147.9
16 + weeks	2092	3165.0(331.1)	2115	3057.9(341.6)	107.1(p = 0.033)	86.8	127.5

Ϯ *P value adjusted for clustering*

We conducted multivariate analyses using a random-effect regression model to adjust for clustering and selected covariates (Table 4). Two models are presented: Model 1 adjusts only for clustering, and Model 2 for clustering and selected covariates that include newborn sex, the timing of first ANC visit, women’s age, education, wealth, number of pregnancies, birth to pregnancy interval, MUAC, and number of ANC visits. After adjusting for clustering in Model 1, birth weight was significantly higher in the intervention arm than in the control arm (ß coef. = 122.4; p = 0.006). Of note, the regression coefficient in this model represents the adjusted mean difference in BW between the two arms. Adjusting for selected covariates in Model 2 did not modify the effect of the intervention on birth weight, with a statistically significant regression coefficient of 114.3 (p = 0.011).

**Table 4:** Estimated regression coefficients from the MLE random effects regression model to show the effect of the ENAT intervention on birth weight: Model 1: Adjusted for clustering, Model 2: Adjusted for clustering and selected covariates

	Model 1 *(Adjusted for clustering)*		Model 2 *(Adjusted for clustering and selected covariates* Ϯ*)*	
	**ß coefficient**	**P value**	**ß coefficient**	**P value**
Study arm				
Control (reference category)	0.0		0.0	
Intervention	122.4	0.006	114.3	0.011
Intercept	3032.7	0.000	2960.4	0.000

Ϯ *adjusted for newborn sex, timing of first ANC visit, women’s age, education, wealth, number of pregnancies, birth to pregnancy interval, MUAC, number of ANC visits*

#### Low birth weight (LBW)

In Table 5, we present the incidence rate of LBW for the total sample stratified by newborn sex, mother’s age and gestational age at the first ANC visit. The data show that the incidence of LBW was 4.7% and 7.3% in the intervention and control arms, respectively. Compared to the control arms, LBW was 35.6% lower in the intervention arm, and the difference between the two arms was statistically significant (p = 0.018). A lower incidence of LBW was noted in the intervention arm compared to the control for the male babies (4.7% vs. 7.0%, p = 0.041) as well as the female babies (4.7% vs. 7.6%, p = 0.055). The analysis by mother’s age also revealed lower LBW rates in the intervention arm than in the control arm across all age brackets. The subgroup analysis by gestational age at the first ANC visit also revealed a significantly lower incidence of LBW in the intervention arm than in the control arm irrespective of the timing of the first ANC visit. In the intervention arm, the births whose mothers initiated the first ANC visit during the first trimester of pregnancy appeared to have a significantly lower incidence of LBW at 5.1% compared to 8.7% for the same in the control arm (p = 0.048). We also found a reduction of 35.4% in the incidence of LBW in the intervention arm compared to the control among newborns whose mothers had their first ANC visit later than the first trimester.

**Table 5:** The incidence of low birth weight (LBW), absolute difference in LBW between the intervention and control arm, and percent reduction in LBW by study arm

	Intervention		Control		P value Ϯ	% Reduction in LBW
	**n**	**LBW %**	**n**	**LBW%**		
All sample	3445	4.7	3192	7.3	0.018	35.6%
Newborn sex						
Male	1773	4.7	1647	7.0	0.041	32.8%
Female	1672	4.7	1545	7.6	0.055	38.1%
Age of mother						
15–19	218	5.5	241	7.9	0.310	30.4%
20–24	1056	5.2	959	8.4	0.059	38.1%
25–34	1827	4.2	1709	6.6	0.002	36.3%
35–49	344	5.2	282	7.1	0.336	26.8%
Gestational age at first visit						
< 16 weeks	1249	5.1	1054	8.7	0.048	41.4%
16 + weeks	2092	4.3	2115	6.5	0.049	35.4%

Ϯ *P value adjusted for clustering*

Multivariate analyses using a random effects logit model further confirmed that babies in the control arms had significantly higher LBW than their counterparts in the intervention even after adjusting for clustering and selected covariates. We present two models in Table 6. Model 1 adjusted only for clustering and found a significantly lower relative risk (RR) of 0.61 (p = 0.005) that was associated with the intervention arm, which suggests that the risk of LBW decreased by 39% in the intervention arm compared to the control. In Model 2, we adjusted for clustering and several covariates, including newborn sex, the timing of the first ANC visit, women’s age, education, wealth, number of pregnancies, birth to pregnancy interval, MUAC, and number of ANC visits. The adjusted RR associated with the intervention arm was 0.66 (p = 0.030), which was statistically significant.

**Table 6:** Estimated regression coefficients from the random effects logit model to show the effect of the ENAT intervention on low birth weight (LBW): Model 1: Adjusted for clustering, Model 2: Adjusted for clustering and selected covariates

	Model 1 (Adjusted for clustering)		Model 2 (Adjusted for clustering and selected covariates Ϯ)	
	Risk Ratio	P value	Risk Ratio	P value
Study arm				
Control (reference category)	1.0		1.0	
Intervention	0.61	0.005	0.66	0.030
Intercept (probability)	0.070	0.000	0.091	0.000

Ϯ *adjusted for newborn sex, first ANC visit, women’s age, education, wealth, number of pregnancies, birth to pregnancy interval, MUAC, number of ANC visits*

## Discussion

This study presented findings indicating that newborns from mothers who received the ENAT intervention exhibited significantly higher birth weights in comparison to newborns from mothers who did not receive the intervention. We found an effect size of 108 g for mean birth weight and a 36% reduction in LBW in the intervention arm compared to the control arm. These findings were further confirmed by multivariate analyses that adjusted for clustering and selected covariates.

Available studies on the relationship between birthweight and the quality of ANC services suggested a lower risk of LBW associated with high-quality ANC services [30–38]. However, most of these studies lack rigor due to their cross-sectional nature, and only a few utilized data from case-control designs. Irrespective of the nature and contents of the intervention, a prospective randomized controlled clinical effectiveness trial that tested the impact of a combination of nutritious supplementary food and several proven chemotherapeutic interventions to control common infections in Sierra Leone found an effect size of 70 g birth weight and 0.3 cm length [39]. Others also reported improved birth weight with nutrition supplementation and nutrition education on top of routine ANC services in different settings [40–42]. A systematic review found a decreased LBW rate associated with oral supplementation to pregnant women, including vitamin A, low-dose calcium, zinc, multiple micronutrients, and nutritional education [43].

The ENAT intervention strengthened the quality of existing routine ANC-based services in public health centers through training health workers, gap filling, mentoring and supportive supervision, and data use. Most importantly, ENAT has improved infection prevention in pregnant women by strengthening and providing point-of-care testing in health centers, which otherwise are not always available in public health facilities despite the national ANC guideline recommendations [19]. Data on the ANC contents we reported here in particular divulged higher coverage for the screening of ASB, sexually transmitted infections, and the provision of deworming in the intervention facilities than in the control. We also found higher coverage in the intervention health facilities for other services, such as screening for hemoglobin and urinalysis. Among other factors, we can posit that screening and detection of ASB and sexually transmitted infections and treating such infections promptly during pregnancy may have an important role in the recorded outcome. Studies have reported that early detection and treatment of maternal infections, such as ASB, have improved birth weights and other birth outcomes [44], while untreated bacterial infection leads to low birth weight [45]. Likewise, several studies and meta-analyses found a higher risk of low birth weight with sexually transmitted infections during pregnancy [46–48]. Another important finding worth noting is the higher coverage of deworming in the ENAT health centers compared to the control (49.6% vs. 8.1%). There is a general dearth of data on the relationship between deworming and birthweight; however, a recent study that analyzed data from 95 countries found an 11% reduction in the odds of low birth weight for women receiving deworming medicine [49]. The WHO recommends periodic deworming of pregnant women after the first trimester [50].

The main strength of this study was the use of a CRCT design with adequate statistical power that allowed for the detection of changes in the primary and secondary outcomes. Second, we tested whether the pregnant women who participated in the intervention and control arms were similar in terms of selected background characteristics, and this analysis found no statistically significant differences between the two arms in terms of participants’ age, education, household wealth, number of pregnancies, number of living children, birth-to-pregnancy interval, maternal weight, MUAC and the gestational age at first ANC booking. Indeed, the balance between study arms of observed and unobserved factors is a goal of randomization for unbiased estimation of intervention effects in a CRCT design [36]. Third, efforts were made to ensure the quality of birth weight data by providing new weight scales, training to health workers, and supportive supervision in the study health centers. These important data quality steps undoubtedly resulted in fairly good quality birth weight data with a substantial decrease in heaping compared to previously available birth weight data in the country. Provision of ANC services and measuring birth weight are routine tasks for staff in the study health centers, and this study did not interfere with the day-to-day routine services of the health facilities. This should be emphasized as another strength of this study.

Nevertheless, this study was not without limitations. There were 29.2% of the pregnant women in the intervention and 33.7% in the control arms who did not have birth weight data and were thus considered lost to follow-up. However, this study was powered considering a 41% loss to follow-up and achieved more than the anticipated sample size for birth weight in both study arms. We assessed for any selection bias associated with lost to follow-up by comparing the enrollment characteristics of the pregnant women who were the basis for the final analysis and those participants originally recruited and lost to follow-up. Those with the outcome and those lost to follow-up were broadly similar in terms of several enrollment characteristics, suggesting that the main outcome was unlikely to be biased by the lost to follow-up of the participants. However, we could not rule out the presence of unmeasured factors that could vary between those with the outcome and those lost to follow-up. Our study lacked data on the pregnant women’s health status at their ANC booking and during follow-up, and there were no detailed and organized data that indicated the results of the screening tests/diagnosis, the types of treatments provided as well as the outcomes of the treatments received. This information is crucial to examine how the different components of ANC services influence the recorded birthweight. Another limitation that should be acknowledged is that apart from the birth weight data, there was no similar quality assurance or monitoring of the large array of ANC data that were extracted from the ANC and delivery registration books because this study could not interfere with the routine activities of the health centers. Nonetheless, we assume that any data quality issues related to the uptake of ANC services are randomly distributed between both study arms, without any systematic relationship to bias the findings.

## Conclusion

The findings of this study provide sufficient evidence of the effectiveness of the ENAT intervention in improving birth weight in the study population. ENAT is likely to reverberate with maternal and newborn health programmers because it has demonstrated that existing routine ANC packages in public facilities can improve birth weight further when implemented fully and appropriately with particular emphasis on point-of-care testing and infection prevention. The intervention is easier to implement and scalable within the ANC framework of public health facilities. However, it costs more than the Ethiopian government’s funded ANC services that are freely available to pregnant women in health centers.

## Data Availability

The raw data used for this study can be available upon request. Point of contact: Dr. Yared Mekonnen, Mela Research, P.O. Box 34,422, Addis Ababa, Ethiopia; E-mail: yared@melareserach.com.

## List of Abbreviations

ANC: Antenatal Care
ASB: Asymptomatic Bacteriuria
BW: Birth Weight
CI: Confidence Interval, CRCT, Cluster Randomized Controlled Trial
EG: Ethiopia Government
ENAT: Nutrition and Antenatal Infection Treatment
HC: Health Centre
ICC: Interclass Correlation Coefficient
IFA: Iron and Folic Acid
IRB: Institute Review Board
LBW: Low Birth Weight
LTFU: Loss to follow up
MOH: Ministry of Health
MUAC: Mid Upper Arm Circumference
PHU: Primary Health Care Unit
PoC: Point of Care
RHB: Regional Health Bureau
SD: Standard Deviation
TTI: Tetanus Toxoid Injection
WHO: World Health Organization
